# Lo moderno, lo indígena, lo mental: institucionalización de la psiquiatría en Perú, 1859-1947

**DOI:** 10.1590/S0104-59702024000100071

**Published:** 2024-12-16

**Authors:** Aida Alejandra Golcman

**Affiliations:** i Profesora-investigadora, Universidad Nacional de Tucumán. San Miguel de Tucumán – Tucumán – Argentina alejandragolcman@gmail.com

Este libro narra el proceso de institucionalización y profesionalización de la psiquiatría en Perú a partir de una reconstrucción de la disciplina desde diversas rutas posibles: instituciones hospitalarias, casos judiciales, discursos científicos, personajes destacados, tratamientos y políticas de salud mental. Esta manera de trabajar a partir de diferentes nodos permite pensar la psiquiatría como dispositivo estructurante de espacios más allá de la práctica clínica. Así, la obra presenta un modelo de análisis posible para un campo completo, en este caso el de un país.

Es un libro escrito en diálogo permanente con países vecinos y con el contexto latinoamericano en general, que se apoya tanto en los proyectos editoriales previos del autor (Ríos Molina, Ruperthuz, 2022) como en otros aportes historiográficos de los últimos años que se hicieron preguntas acerca de toda la región (Campos, Ruperthuz, 2022; Ordorika, Golcman, 2021). Este rasgo le provee características particulares al libro. Este rasgo le provee características particulares al libro. Con la ilusión de encontrar exclusividad en sus características ya que no es lo mismo encarar su investigación conociendo su entorno que mirar un objeto de estudio aislado – en este caso la psiquiatría en Perú. De este modo, es un texto que se acerca a los nuevos desafíos en la historia cultural de la psiquiatría latinoamericana: por una parte, encontrar las lógicas propias de la región, las cercanías y las distancias, las similitudes y las diferencias, y, por otra parte, comprender que, para pensarnos en las coordenadas de la historia global, primero debemos detenernos en un entendimiento regional.

En el Perú decimonónico teñido por ideas liberales y fuerte control social, el texto analiza el proceso de profesionalización de la psiquiatría, algunos de sus protagonistas relevantes, y su relación con la comunidad científica internacional. Además, describe los cambios en las instituciones psiquiátricas, los vínculos de poder forjados y la diversidad de voces que se escucharon en estos espacios – familiares, monjas, policías, jueces, abogados etc. Al mismo tiempo, el autor describe el mundo de la psiquiatría peruana como un “espacio de modernidad sin modernización” (Ríos Molina, 2023, p.16), ya que los proyectos europeizantes de reformas que mejoren la vida de pacientes se veían constantemente opacados por los clásicos males endémicos de la psiquiatría americana: la falta de presupuesto y el hacinamiento.

El autor reconstruye la clínica psiquiátrica en Perú desde una lógica temporal que se repite en toda la región donde se combinan la llegada de diversas tecnologías (por ejemplo, las terapias de shock) y la circulación internacional de personajes ilustres. Para el caso peruano se destacó el emblemático Honorio Delgado y, a partir de su derrotero, el lugar que le cupo al psicoanálisis como una primera respuesta posible para la clínica de la demencia precoz.

Otro trazo notable de la obra es la puesta en valor de la figura de Víctor Larco Herrera y su papel fundamental para el devenir de la psiquiatría peruana (Ríos Molina, 2023, p.89). Dos son los puntos para enfatizar sobre dicho personaje. Por un lado, el autor muestra cómo la acción de este empresario deja de manifiesto lo definitorio de la iniciativa privada en la conformación del campo de la salud mental de Perú ante las carencias estatales. Y, por otro lado, Ríos Molina demuestra que, ante la pregunta de los protagonistas de toda la región sobre cómo llegar a la modernidad psiquiátrica, Larco Herrera sostuvo que dicha modernidad podía generarse en el Sur. Pues, luego de su viaje internacional y su recorrido por las instituciones psiquiátricas europeas más destacadas, él tomó como referencia a seguir el modelo de Domingo Cabred en Buenos Aires. Este análisis del autor abona a la construcción historiográfica que invita a pensar puentes y rasgos regionales y, al mismo tiempo, posiciona al Cono Sur como un faro valioso de saberes y prácticas psiquiátricas.

El capítulo 3, “La tristeza de una raza degenerada: psicología y psicopatología del indio”, es el corazón del libro, y su esencia se disemina en los capítulos siguientes. Es una lectura que incluye las preguntas por las otredades, que se puede articular con los debates actuales sobre las diversidades y que plantea el adentro y el afuera de la “occidentalidad” en coordenadas psiquiátricas. La reflexión sobre la psicología y la psicopatología indígena fue lo que destacó a Perú del resto del campo disciplinar regional, y por lo tanto dicho estudio es uno de los grandes aportes de este texto.

Así, Perú fue el único país interesado en la psicología del indígena a partir de lo que se llamó folklor psiquiátrico. El autor explica que se trató de una línea de reflexión muy culturalista que buscaba acumular información como si los indígenas fueran piezas arqueológicas, pero no tenía interés en la especificidad de los casos. Así, a partir de la concepción de Althusser (1988), la psiquiatría fue un saber racista y clasista que funcionó como aparato ideológico del Estado desde su narrativa científica y significó otro camino posible para marcar la superioridad occidental.

De este modo, el libro interpela el lugar de la psiquiatría como agente de los Estados en América Latina y el uso político del padecimiento mental. Esto se plasma también en el capítulo 6 sobre el movimiento de higiene mental, donde se evidencia el interés de psiquiatras por prevenir la degeneración racial: eugenistas que intentan mejorar la raza, educadores que pretenden detectar infancias “anormales” etc. Y también en el capítulo 5 con la descripción de casos judiciales como el de Alejandrino Montes donde graves crímenes cometidos justifican prejuicios raciales en el discurso público.

Este libro acabado, amplio, diverso, permite tener una secuencia de imágenes del campo psiquiátrico peruano y de sus actores entre 1859 y 1947. Su exhaustivo trabajo de fuentes, su distinguido manejo de bibliografía secundaria, sus sugerentes cruces teóricos y sus evocadoras preguntas para pensar más allá de los bordes clínicos convierten al libro inmediatamente en una referencia obligada tanto para la historia cultural de Perú como para la historia latinoamericana de los saberes psi. El texto, por último, exhorta a la historiografía regional a seguir explorando qué versiones de indígenas, pobres, inmigrantes, campesinos, mujeres, infantes dio la psiquiatría a partir de su agencia y cuáles fueron sus efectos sociales, ideológicos, institucionales.
